# A Multi-Component High-Fibre Meal Replacement Containing *Pyrus bretschneideri* Rehd. Pomace Alleviates Diet-Induced Obesity by Modulating the Gut Microbiota, Bile Acid Metabolism and Hepatic AMPK Signalling

**DOI:** 10.3390/foods15193377

**Published:** 2026-09-23

**Authors:** Jie Li, Ying Ye, Fubao Fan, Ziyi Han, Jinyan Liu

**Affiliations:** College of Agriculture and Animal Husbandry, Qinghai University, Xining 810016, China; xli6818@163.com (J.L.); fubaofan@gmail.com (F.F.); 17616063887@163.com (Z.H.); liujinyan0111@163.com (J.L.)

**Keywords:** meal replacement powder, dietary fibre, gut microbiota, AMPK pathway, metabolomics

## Abstract

This study employed an integrated multi-omics approach to investigate the regulatory effects and molecular mechanisms of a multi-component, high-dietary-fibre meal replacement powder containing *Pyrus bretschneideri* Rehd. pomace (PDMR) in high-fat-diet-induced obese mice. The results showed that PDMR significantly reduced body weight gain, Lee’s index, serum lipid and inflammatory cytokine levels, and improved adipocyte morphology. Mechanistically, PDMR activated the AMPK signalling pathway, suppressed lipogenesis via the ACC-SREBP1c-FAS axis, and promoted fatty acid β-oxidation and thermogenesis through the PPARα-CPT-1 and PGC-1α/UCP-1 axes. Moreover, PDMR remodelled the gut microbiota by enriching bacteria associated with SCFA production and anti-inflammatory activity (*Alloprevotella*, *Ruminococcaceae*, *Odoribacter* and *Prevotellaceae_Ga6A1_group*) and inhibiting bacteria associated with pro-inflammatory and fat-accumulation effects (*Candidatus Saccharimonas* and *Clostridia_UCG-014*), whilst reversing the excessive accumulation of secondary bile acids. Correlation analysis established a multi-level regulatory network linking gut microbiota, secondary bile acid metabolites and AMPK target gene expression. Collectively, PDMR may ameliorate lipid metabolic disorders through a potential gut microbiota–secondary bile acid–AMPK axis, providing a scientific basis for the high-value utilisation of *Pyrus bretschneideri* Rehd. and the development of functional meal replacement foods.

## 1. Introduction

With socioeconomic development and changes in human lifestyles, the spectrum of common human diseases has shifted considerably. Obesity, hyperlipidaemia and other metabolic diseases have reached epidemic proportions worldwide, posing a major threat to human health [1]. Long-term consumption of high-fat, high-calorie diets is a key environmental factor inducing obesity. The resultant lipid metabolic disorders are regarded as the core pathological basis of metabolic syndrome, characterised by dyslipidaemia, adipose tissue hypertrophy, ectopic lipid deposition in the liver and a concomitant chronic low-grade inflammatory state; these disorders further increase the risk of chronic diseases such as type 2 diabetes mellitus, non-alcoholic fatty liver disease and cardiovascular diseases [2,3]. Although lipid-lowering drugs such as statins and orlistat are currently available in clinical practice, they are mainly used as medical treatments rather than daily interventions, and most synthetic anti-obesity drugs have certain limitations. For instance, statins are generally well tolerated but are associated with muscle symptoms, elevated liver enzymes, and an increased risk of new-onset diabetes [4]. Glucagon-like peptide-1 (GLP-1) drugs have been shown in preliminary clinical studies to cause nausea in some patients; moreover, certain GLP-1 receptor agonists can affect immunomodulatory function in the elderly, immunocompromised individuals, and those with hepatic impairment, leading to adverse effects [5,6]. Concurrently, multiple studies have reported that weight regain after discontinuation of weight-loss drugs is relatively common, with the rate of regain being particularly pronounced following cessation of the GLP-1 receptor agonist semaglutide [7,8]. Therefore, regulating lipid metabolism through daily dietary interventions to achieve the prevention and adjunctive treatment of metabolic diseases has become a major research focus in the field of food science [9,10].

Dietary fibre is a class of plant-derived polysaccharides that cannot be directly degraded by host digestive enzymes. In recent years, it has attracted considerable attention as a promising nutritional intervention for the prevention and management of obesity [11]. A large body of research has established that dietary fibre intake is significantly inversely associated with the risk of metabolic diseases, and dietary fibre supplementation has become an important strategy for the prevention and control of these conditions [12]. Notably, unlike small-molecule nutrients, the physiological activity of dietary fibre is highly dependent on fermentation by the gut microbiota. The physicochemical properties of dietary fibre, including solubility, monosaccharide composition, glycosidic linkage types, and viscosity, significantly influence the extent to which it is selectively utilised by the gut microbiota and the resulting fermentation patterns [13,14]. For example, fermentable fibres such as pectin can be degraded by the gut microbiota through carbohydrate-active enzymes (CAZymes) and further metabolised via cross-feeding within the microbial community, producing short-chain fatty acids (SCFAs) such as acetate, propionate, and butyrate [15]. SCFAs are not only an important energy source for intestinal epithelial cells but also act as signalling molecules involved in the regulation of lipid metabolism, inflammatory responses, and immune homeostasis, making them key mediators of the gut microbiota–host metabolic interaction axis [16,17]. Furthermore, substantial evidence from animal experiments, epidemiological studies and clinical research indicates that diets rich in dietary fibre can modulate gut microbiota composition, prevent obesity, and reduce body weight and visceral fat deposition [18,19]. In this context, fruit pomace—a by-product of fruit processing—has emerged as a valuable and sustainable source of dietary fibre owing to its richness in fermentable fibre components such as pectin, cellulose, and hemicellulose. Moreover, recent studies have shown that dietary fibre derived from pomace can increase propionate and total SCFA levels in in vitro fermentation and alleviate body weight gain, dyslipidaemia, and adipose tissue hypertrophy in high-fat-diet-induced obese mice [20,21]. Therefore, pomace, as a source of functional dietary fibre, offers the dual benefits of waste valorisation and functional food development.

*Pyrus bretschneideri* Rehd., belonging to the genus *Pyrus* L. in the family Rosaceae, is commonly known as Suanguo (sour pear) and is a characteristic local pear cultivar grown predominantly in Tongren City, Huangnan Tibetan Autonomous Prefecture, Qinghai Province, on the Qinghai–Tibet Plateau. The local government has designated the *Pyrus bretschneideri* Rehd. industry as a characteristic pillar industry, giving it considerable agricultural and economic value. Unlike the better-known *Pyrus communis* L. and *Pyrus pyrifolia* (Burm. f.) Nakai (the latter of which favours warm and humid conditions), *Pyrus bretschneideri* Rehd. exhibits exceptional cold and drought tolerance. In addition, owing to the unique plateau continental climate, characterised by high altitude, low temperatures and marked diurnal temperature variation, the fruit possesses a distinctive flavour profile, being fragrant, juicy, golden-coloured and pleasantly sweet–sour [22]. In addition to its sensory attributes, *Pyrus bretschneideri* Rehd. pomace is abundant in various bioactive components, including soluble and insoluble dietary fibre as the major constituents, together with minor amounts of phenolic compounds such as chlorogenic acid and protocatechuic acid, and flavonoids such as catechin, isoquercitrin, and rutin [23]. These components offer considerable potential for the development of functional foods, and studies have preliminarily confirmed the anti-obesity potential of pear-pomace-derived products. Chen et al. [24] found that three different pectin fractions isolated from pear pomace all exhibited favourable lipid-lowering effects in vitro. Rhyu et al. [25] reported that the aqueous extract of pear pomace reduced body weight gain, body fat accumulation and the expression of lipogenic enzymes in high-fat-diet-fed mice by activating the AMPK pathway; this effect was abolished by the specific AMPK inhibitor compound C in 3T3-L1 adipocytes. Zhao et al. [26] demonstrated that soluble dietary fibre from pear pomace inhibited fat deposition in high-fat-diet-induced obese mice by modulating adipose inflammation and activating the ADPN-AMPK/PPAR-α signalling pathway. Moreover, phenolic compounds present in pear pomace, including chlorogenic acid, coumaric acid and caffeic acid, have been identified as potential stimulators of AMPK phosphorylation. Collectively, these findings establish pear pomace as a rich source of dietary fibre with anti-obesity properties, which may regulate obesity through activation of the AMPK signalling pathway. However, existing studies have mostly focused on the single dimension of the AMPK pathway, and there remains a lack of systematic research on the metabolic signals produced by gut microbiota fermentation of pear-pomace-derived products and how these interact with the AMPK pathway.

Furthermore, although the aforementioned studies provided valuable mechanistic insights, they focus solely on isolated fibre fractions or aqueous extracts of pear pomace, overlooking the fact that, in actual dietary intake, dietary fibre is only one component of a complex food matrix. Meal replacement powders, which are nutritious, convenient daily foods designed to replace one or more meals, offer a practical and sustainable means of dietary fibre intervention. Moreover, recent studies have confirmed the efficacy of meal replacement products in body weight and glycaemic management, and high-dietary-fibre meal replacement formulations have shown considerable potential in ameliorating obesity and lipid metabolic disorders [27,28]. However, current processing of *Pyrus bretschneideri* Rehd. remains primarily focused on juice and jam production; the utilisation of its by-product pomace in the development of functional meal replacement products, as well as the intervention effects and underlying molecular mechanisms of such products, remains largely unexplored.

Accordingly, the present study utilised *Pyrus bretschneideri* Rehd. pomace powder as the primary raw material to formulate a multi-component, high-dietary-fibre meal replacement powder (PDMR). High-fat-diet-induced obese mice were used as the experimental model, and the lipid-lowering effects of PDMR were comprehensively evaluated across multiple dimensions, including body weight, serum biochemical parameters, histopathological examination, molecular pathway analysis, 16S rRNA gut microbiota sequencing and untargeted metabolomics; the underlying mechanisms by which PDMR regulates lipid metabolic disorders were further elucidated. This study aims to provide a theoretical basis for the high-value utilisation of the characteristic *Pyrus bretschneideri* Rehd. resource from the Qinghai–Tibet Plateau and to provide theoretical support for the development of high-dietary-fibre functional foods.

## 2. Materials and Methods

### 2.1. Materials and Reagents

*Pyrus bretschneideri* Rehd. fruits were collected from the Tongren region, Qinghai Province, China (35°31′ N, 102°01′ E), and authenticated by Professor Ying Ye of Qinghai University according to the *Flora of Qinghai*. Soy protein isolate was purchased from Linyi Shansong Biological Products Co., Ltd., Linyi, China; konjac flour was purchased from Bozhou Huazhitang Biotechnology Co., Ltd., Bozhou, China; aspartame was purchased from Jiangsu Vitasweet Co., Ltd., Liyang, China; xylitol was purchased from Shandong Futian Pharmaceutical Co., Ltd., Dezhou, China; assay kits for serum lipid levels (TG, Cat. No. A110-1-1; TC, Cat. No. A111-1-1; LDL-C, Cat. No. A113-1-1; HDL-C, Cat. No. A112-1-1; FFA, Cat. No. A042-1-1; all lot numbers: 20250624) were purchased from Nanjing Jiancheng Bioengineering Institute Co., Ltd. (Nanjing, China); assay kits for inflammatory cytokines (TLR4, Cat. No. HP-E30625; IL-6, Cat. No. HP-E20012; IL-1β, Cat. No. HP-E20533; all lot numbers: 202505) were purchased from Shanghai Hepeng Biotechnology Co., Ltd. (Shanghai, China); and orlistat was purchased from Chongqing Huasen Pharmaceutical Co., Ltd. (Chongqing, China).

### 2.2. Preparation of the PDMR

PDMR was prepared using *Pyrus bretschneideri* Rehd. pomace powder as the main ingredient, aspartame and xylitol as sweeteners, konjac flour as a thickening agent, and soy protein isolate as a filler. The formulation was optimised through single-factor and response surface experiments, and the final formulation was as follows: 42% *Pyrus bretschneideri* Rehd. pomace powder, 0.2% aspartame, 11% konjac flour, 10% xylitol and 36.8% soy protein isolate. The total dietary fibre content of the meal replacement powder was determined to be (56.19 ± 0.60)%, with soluble dietary fibre at (8.36 ± 0.25)% and insoluble dietary fibre at (44.61 ± 0.70)%.

### 2.3. Animal Experimental Design

A total of 60 specific pathogen-free (SPF) adult male mice (5 weeks old, body weight 18–22 g) were obtained from Lanzhou Veterinary Research Institute (Laboratory Animal Centre, Chinese Academy of Agricultural Sciences, Lanzhou, China), with quality certificate number SCXK (Gansu) 2020-0002. All animal procedures were conducted in strict accordance with the National Institutes of Health Guide for the Care and Use of Laboratory Animals and were approved by the Animal Ethics Committee of Qinghai University (approval number: SL-2023011). The mice were housed under standard laboratory conditions, with an ambient temperature of 22–25 °C, humidity of 50 ± 5%, and a 12 h light/dark cycle.

After one week of adaptive feeding, the mice were randomly divided into six groups (*n* = 10 per group; sample size determined on the basis of previous experience from our research group and relevant literature [29,30]): a negative control group (NC), a model control group (MC), a positive control group (PC, orally administered 60 mg/kg/day orlistat at a fixed dosing volume of 0.1 mL/10 g body weight), and low-, medium- and high-dose meal replacement powder groups (LMR, MMR and HMR, respectively). The NC group was fed a standard maintenance diet (product number: XT101WC-008, Jiangsu Xietong Pharmaceutical Bioengineering Co., Ltd., Nanjing, China); the MC and PC groups were fed a high-fat diet consisting of 5% egg yolk powder, 10% lard, 1% cholesterol, 4% milk powder, 0.1% sodium cholate and 79.9% standard diet; and the LMR, MMR and HMR groups were fed high-fat diets supplemented with different doses of PDMR: low-dose PDMR high-fat diet (10% PDMR), medium-dose PDMR high-fat diet (20% PDMR) and high-dose PDMR high-fat diet (30% PDMR). The experiment lasted 28 d, during which the mice had ad libitum access to food and water. Body weight was measured every 3 d, and cages were rotated daily to minimise environmental confounding.

After 28 d, the mice were fasted for 12 h with free access to water. They were then anaesthetised by isoflurane inhalation, induced with 3–5% isoflurane and maintained with 1.5–2.5% isoflurane at an oxygen flow rate of 1 L/min. Once the righting reflex and pain reflex had disappeared, body weight and body length (naso-anal length) were measured. Anaesthesia was maintained for approximately 10 min, during which body length and body weight measurements and retro-orbital venous plexus blood collection were completed. Blood was collected, and serum was separated and stored at −80 °C. Subsequently, the mice were euthanised by cervical dislocation under deep anaesthesia and immediately dissected. Major organs and tissues were weighed and recorded; white adipose tissue, brown adipose tissue, liver tissue and caecal contents were aliquoted and stored at −80 °C. This study was not blinded, and no animals were excluded during the experiment. Serum biochemical parameters were assessed using all animal samples (*n* = 10 per group). For histopathological examination (H&E staining), Western blotting, RT-qPCR, and 16S rRNA gene sequencing, three animals per group were randomly selected; for untargeted metabolomics, six animals per group were randomly selected.

### 2.4. Determination of Organ Index and Lee’s Index

The organ index and Lee’s index were calculated according to Equations (1) and (2), respectively, as follows:

(1) $$Organ\;index\left(\%\right)=\frac{organ\;weight}{body\;weight}\times 100\%$$

(2) $$\text{Lee’s}\;index=∛\frac{w}{l}$$

where w is the body weight of the mouse (g) and l is the naso-anal length of the mouse (cm).

### 2.5. Blood Biochemical Analysis

Serum levels of TG, TC, LDL-C, FFA and HDL-C, as well as the inflammatory cytokines IL-6, IL-1β and TLR4, were measured using commercially available assay kits according to the manufacturer’s instructions.

### 2.6. Histological Analysis

Epididymal adipose tissues from the mice were fixed in 4% paraformaldehyde for 12 h, embedded in paraffin, and serially sectioned at a thickness of 4 μm using a rotary microtome (RM2016, Leica, Wetzlar, Germany). The sections were then subjected to H&E staining using a staining kit (C2103, Wuhan Baiqiandu Biotechnology Co., Ltd., Wuhan, China) and mounted with neutral resin. Finally, the stained sections were observed and images were captured using a digital imaging system (DS-U3, Nikon, Tokyo, Japan) [31].

### 2.7. Western Blot and RT-qPCR Analysis

The expression levels of the genes encoding CPT-1, PPARα, FAS, PGC-1α and UCP-1 in mouse adipose tissue were determined. Total RNA was extracted from mouse adipose tissue using TRIpure Total RNA Extraction Reagent (EP013, ELK Biotechnology, Wuhan, China). RNA concentration and purity were measured using an ultra-micro UV-Vis spectrophotometer (ND-100, Hangzhou Mio Instrument Co., Ltd., Hangzhou, China). First-strand cDNA was synthesised using an EntiLink™ 1st Strand cDNA Synthesis Kit (EQ003, ELK Biotechnology, Wuhan, China) on a gene amplification instrument TC-XP (Hangzhou Bioer Technology, Hangzhou, China) according to the manufacturer’s instructions. RT-qPCR was performed using a QuantStudio 6 Flex Real-Time PCR System (Life Technologies, Carlsbad, CA, USA) and EnTurbo™ SYBR Green PCR SuperMix (EQ001, ELK Biotechnology, Wuhan, China). Thermal cycling conditions were as follows: pre-denaturation at 95 °C for 3 min; 40 cycles of 95 °C for 10 s, 58 °C for 30 s, and 72 °C for 30 s; followed by melting curve analysis. The primer sequences for CPT-1, PPARα, FAS, PGC-1α, UCP-1, and GAPDH are shown in Table 1. GAPDH was used as the internal reference gene for normalisation. Relative gene expression was calculated using the 2^−ΔΔCt^ method.

The protein expression levels of p-AMPK, p-ACC and SREBP1c in mouse liver tissue were determined. The liver tissues were washed with pre-cooled PBS (AS1025, ASPEN, Beijing, China), cut into small pieces, and homogenised in RIPA total protein lysis buffer (AS1004, ASPEN, China) containing protease inhibitor cocktail (04693159001, ROCHE, Basel, Switzerland), 100× PMSF (AS1006, ASPEN, China), and phosphatase inhibitor (AS1008, ASPEN, China). The homogenised tissue suspension was lysed on ice and centrifuged (12,000 rpm, 30 min, 4 °C) using a refrigerated centrifuge (HSC-2015L, Scientz, Ningbo, China). The protein concentration was quantified using a BCA protein assay kit (AS1086, ASPEN, China). Equal amounts of protein were mixed with 5× protein loading buffer and denatured using a dry block heater (DTH-100, Hangzhou Mio Instrument, Hangzhou, China). Proteins were separated by SDS-PAGE on a vertical electrophoresis system (DYY-6C and DYCZ-24DN, Beijing Liuyi Instrument Factory, Beijing, China), with a 10% separating gel and a 5% stacking gel. Proteins were transferred to a 0.45 µm PVDF membrane (IPVH00010, Millipore, Billerica, MA, USA) using a transfer electrophoresis tank (DYCZ-400D, Beijing Liuyi Instrument Factory, Beijing, China). After blocking with 5% skimmed milk powder in TBST for 3 h at room temperature, the membrane was cut and incubated overnight at 4 °C with shaking with primary antibodies diluted in primary antibody dilution buffer (AS1061, ASPEN, China). After washing, the membrane was incubated with HRP-conjugated secondary antibodies diluted in secondary antibody dilution buffer (AS1058, ASPEN, China) for 30 min at room temperature. The signal was visualised using an ECL chemiluminescence detection kit (AS1059, ASPEN, China) and exposed to medical X-ray film (Super RX-N, Fujifilm, Tokyo, Japan) in a cassette (AX-, Guangdong Yuehua Medical Instrument Factory, Shantou, China). The film was scanned using a scanner (Perfection V850 Pro, Epson, Suwa, Japan), and the optical density values of the target bands were analysed using the AlphaEaseFC system (Version 4.0).

### 2.8. Gut Microbiota Analysis

The microbial communities in the caecal contents were analysed by 16S rDNA amplicon sequencing. After genomic DNA was extracted from the mouse caecal contents, DNA integrity was assessed by 1% agarose gel electrophoresis. Specific primers with barcodes were synthesised for the designated sequencing region, and PCR amplification was performed using TransStart Fastpfu DNA Polymerase (TransGen AP221-02, TransGen Biotech, Beijing, China). To ensure the accuracy and reproducibility of subsequent data analysis, the minimum number of amplification cycles was determined through preliminary experiments using representative samples, and all samples were amplified with a uniform number of cycles. The amplification products were verified by 1% agarose gel electrophoresis to confirm the expected target band size and then purified using the Agencourt AMPure XP nucleic acid purification kit (Beckman Coulter, Brea, CA, USA). A next-generation high-throughput sequencing library was constructed from the purified products through sequential steps including Y-shaped adapter ligation, magnetic bead-based selection to remove adapter self-ligation fragments, PCR amplification to enrich the library templates, and sodium hydroxide denaturation to generate single-stranded DNA fragments. Sequencing was subsequently performed on a next-generation high-throughput sequencing platform using a sequencing-by-synthesis strategy [32].

### 2.9. Non-Targeted Metabolomics Analysis

Caecal contents were thawed at 4 °C, and an aliquot of 50–100 mg was accurately weighed into a centrifuge tube. After adding 200 μL of pre-cooled water, the sample was vortexed for 60 s. Subsequently, 0.8 mL of pre-cooled methanol/acetonitrile (1:1, *v*/*v*) was added, followed by another 60 s of vortexing. The mixture was subjected to low-temperature ultrasonic extraction for 30 min and then kept at −20 °C for 1 h to precipitate proteins. After centrifugation at 12,000 rpm for 10 min at 4 °C, the supernatant was collected and vacuum-dried. The dried residue was reconstituted in 200 μL of 30% acetonitrile, vortexed, and centrifuged again at 14,000 rpm for 15 min at 4 °C. The supernatant was used for UPLC-HRMS analysis. Sample preparation and LC-MS/MS data acquisition were performed by Jiangsu Sanshu Biotechnology Co., Ltd. (Nantong, China).

The analytical system consisted of a Vanquish UPLC (Thermo, Waltham, MA, USA) coupled to a Q Exactive HFX high-resolution mass spectrometer (Thermo, USA). Chromatographic separation was performed on a Waters HSS T3 column (100 × 2.1 mm, 1.8 μm). Mobile phase A was ultrapure water containing 0.1% formic acid, and mobile phase B was acetonitrile containing 0.1% formic acid. The flow rate was 0.3 mL/min, the column temperature was 40 °C, and the injection volume was 2 μL. The gradient program was as follows: 0–1 min, A/B = 100:0; 4 min, A/B = 40:60; 6.5 min, A/B = 5:95; 6.6–8.0 min, A/B = 100:0. During analysis, samples were maintained at 4 °C in the autosampler. To minimize signal drift, samples were analysed in a random order, and QC samples were evenly inserted throughout the sequence to monitor system stability and data reliability [33].

### 2.10. Statistical Analysis

Data are expressed as mean ± standard deviation (SD). The Shapiro–Wilk test was used to assess the normal distribution of the data. For normally distributed data, one-way analysis of variance (ANOVA) was used for multi-group comparisons, followed by Tukey’s test for post hoc multiple comparisons. Statistical analysis and graphing were performed using GraphPad Prism 9.0 (GraphPad Software, San Diego, CA, USA). A *p*-value < 0.05 was considered statistically significant, and a *p*-value < 0.01 was regarded as highly significant.

## 3. Results

### 3.1. Effects of PDMR on Body Weight, Lee’s Index and Organ Indices in Mice

Body weight monitoring during the 28 d feeding period (Figure 1A) showed that body weight increased rapidly in all groups during the first 7 days, then tended to plateau from days 7 to 22, and remained stable or declined slightly from day 22 onwards. At the end of the feeding period, the body weight gain of mice in the MC group (13.89 ± 3.54 g, Figure 1B) was significantly higher than that of all other groups (*p* < 0.05), indicating that the high-fat-diet-induced obese mouse model was successfully established.

Lee’s index, calculated from body weight and body length, is used to assess the degree of obesity in mice [34]. Calculation of Lee’s index for each group (Figure 1C) revealed that the MC group had a significantly higher value than all other groups (*p* < 0.05), whereas no significant differences were observed among the PDMR intervention groups and the PC group (*p* > 0.05). Furthermore, calculation of organ indices (Table 2) showed that there were no significant differences among groups for the heart, kidney and lung indices (*p* > 0.05). For the liver and spleen indices, the MC group was significantly higher than the other groups (*p* < 0.05), whereas each PDMR intervention group was significantly lower than the MC group (*p* < 0.05) and did not differ significantly from the NC group. These results indicate that the obesity induced by the high-fat diet in mice could be effectively ameliorated by PDMR intervention, with parameters returning to normal levels.

### 3.2. Effects of PDMR on Serum Lipid Levels and Inflammatory Cytokines in Mice

As shown in Figure 2A–E, compared with the NC group, the MC group exhibited significantly elevated levels of TG, TC, LDL-C and FFA (*p* < 0.05), whilst HDL-C was significantly decreased (*p* < 0.05), indicating that the high-fat diet induced severe dyslipidaemia in the mice. After intervention with orlistat and various doses of PDMR, these abnormalities were effectively alleviated. Specifically, compared with the MC group, the low-, medium- and high-dose PDMR groups showed significantly reduced levels of TG, TC, LDL-C and FFA (*p* < 0.05), and HDL-C levels increased by 5.93%, 6.45% and 14.22%, respectively, suggesting that PDMR exhibited a certain dose-dependent improvement in dyslipidaemia.

To investigate whether PDMR intervention could ameliorate lipid metabolic disorders by inhibiting inflammatory pathways, the serum levels of the inflammatory markers IL-6, IL-1β and TLR4 were also measured (Figure 2F–H). Compared with the NC group, the MC group showed significantly elevated levels of IL-6, IL-1β and TLR4 (*p* < 0.05), indicating that the high-fat diet elicited a marked systemic inflammatory response. Compared with the MC group, the PC and all PDMR intervention groups exhibited highly significant reductions in IL-6, IL-1β and TLR4 levels (*p* < 0.01). Notably, the decreases observed in the MMR and HMR groups were even greater than those in the PC group, indicating that PDMR intervention effectively reversed the high-fat-diet-induced inflammatory state in a dose-dependent manner, with the MMR and HMR groups achieving superior improvements to those of orlistat.

### 3.3. Effects of PDMR on Histopathology in Mice

High-fat diets typically lead to adipocyte hypertrophy. In the present study, H&E staining of epididymal adipose tissue (Figure 2I) revealed that, compared with the NC group, adipocytes in the MC group were markedly hypertrophic with abnormal morphology, and the number of cells per unit area was clearly reduced. Following PDMR intervention, the adipocyte morphology in the different dose groups was substantially improved, with the HMR group showing the greatest improvement; its cell morphology was most similar to that of the NC group, and the number of cells per unit area was also higher than that in the PC group.

### 3.4. Effects of PDMR on Protein and mRNA Expression in Mouse Liver

To investigate the potential molecular mechanisms by which PDMR regulates lipid metabolism in obese mice, we examined the activation status of the AMPK signalling pathway and the expression of its key downstream targets in the liver. Western blot analysis (Figure 3A) revealed that the protein expression level of p-AMPKα in the liver was significantly higher in the HMR group than in the MC group (*p* < 0.05), indicating that PDMR intervention effectively induced the phosphorylation and activation of the AMPK pathway. Activated AMPK can directly inactivate its downstream target acetyl-CoA carboxylase (ACC) through phosphorylation, whilst simultaneously suppressing the expression of sterol regulatory element-binding protein 1c (SREBP1c), a key transcription factor in lipid synthesis [35]. Consistent with this, our data showed that, compared with the MC group, the protein expression level of p-ACC in the liver of HMR mice was significantly elevated (*p* < 0.05), whilst the protein expression of SREBP1c and the mRNA level of its target gene fatty acid synthase (FAS) (Figure 3B) were significantly down-regulated (*p* < 0.05).

Furthermore, we examined the mRNA expression of peroxisome proliferator-activated receptor alpha (PPARα), a core transcription factor regulating fatty acid oxidation, and its downstream target carnitine palmitoyltransferase 1 (CPT-1) (Figure 3C,D). Compared with the MC group, the mRNA expression levels of PPARα and CPT-1 in the HMR group were highly significantly up-regulated (*p* < 0.01), suggesting that PDMR intervention could accelerate the entry of fatty acids into mitochondria for β-oxidation. Notably, the mRNA expression of peroxisome proliferator-activated receptor gamma coactivator 1-alpha (PGC-1α) and uncoupling protein 1 (UCP-1) (Figure 3E,F) was also highly significantly up-regulated in the HMR group (*p* < 0.01), indicating that PDMR intervention could promote mitochondrial biogenesis and uncoupled thermogenesis, increase energy expenditure, and further reduce lipid deposition. Collectively, these results reveal that PDMR suppresses fatty acid synthesis by activating the AMPK-ACC-SREBP1c axis, whilst promoting fatty acid β-oxidation and energy expenditure through the AMPK-PPARα-CPT-1 and PGC-1α/UCP-1 axes, thereby bidirectionally regulating metabolic disorders in obese mice.

### 3.5. Effects of PDMR on Gut Microbiota in Mice

#### 3.5.1. Effects of PDMR on Gut Microbial Community Structure

To investigate the overall effect of PDMR on the gut micro-ecology, the structure and composition of the gut microbial community in mice were characterised using 16S rRNA sequencing technology. The Venn diagram (Figure 4A) shows that the total number of OTUs shared among all groups was 214. The numbers of unique OTUs in the NC and MC groups were similar (79 and 76, respectively); taken together with the obese phenotype of the MC group, this suggests that the high-fat diet may not have directly eliminated gut commensals, but rather altered the gut microbiota composition by replacing microbial species. Notably, the HMR group had the highest number of unique OTUs (96), suggesting that the HMR intervention may have introduced novel species.

Subsequently, alpha diversity indices (Figure 4B–D) were used to evaluate microbial richness and diversity. In contrast to the Venn diagram results for the MC group, the Chao1 and ACE indices showed that species richness in the MC group was significantly lower than that in the NC group (*p* < 0.05), which corroborates our speculation that a high-fat diet does not simply reduce the number of species. Furthermore, the HMR group highly significantly reversed the decline in the Chao1 and ACE indices (*p* < 0.01), which was consistent with the Venn diagram results for the HMR group. The trends in the Shannon index (increases of 0.34%, 10.08% and 17.31% in the LMR, MMR and HMR groups, respectively, relative to the MC group) indicated that PDMR intervention had a positive restorative effect on gut microbiota diversity in a dose-dependent manner.

To further assess the overall dispersion of community structure, beta diversity was analysed (Figure 4E,F). Principal coordinate analysis (PCoA) based on Bray–Curtis distance revealed clear clustering of the groups (PCoA1 = 21.35%, PCoA2 = 15.92%). In addition, samples from the NC and MC groups were clearly separated in the ordination space, confirming that the high-fat diet induced a shift in the overall community structure. The samples from all PDMR intervention groups were not only clearly separated from the MC group but also clustered closely towards the NC group, indicating that PDMR intervention effectively ameliorated the disruption of gut microbiota structure caused by the high-fat diet.

#### 3.5.2. Effects of PDMR on Gut Microbial Community Composition

To further investigate the effects of PDMR intervention on the gut microbiota of mice, the microbial community composition was analysed. At the phylum level (Figure 5A), *Firmicutes*, *Bacteroidota* and *Desulfobacterota* were the dominant phyla across all groups, collectively accounting for more than 90% of the community abundance. The *Firmicutesto-Bacteroidota* ratio (F/B) is widely regarded as an important indicator of the overall balance of the gut microbial community, with a higher F/B ratio implying greater efficiency of energy harvest from food and an elevated risk of obesity [36]. Comparison of the F/B ratios among groups (Figure 5B) revealed that the MC group exhibited a substantial increase of 35.69% relative to the NC group, manifesting as a marked enrichment of *Firmicutes* and a concomitant reduction in *Bacteroidota*. PDMR intervention effectively reversed this trend, with the F/B ratios in the LMR, MMR and HMR groups decreasing by 68.50%, 50.16% and 53.64%, respectively, compared with the MC group. At the genus level (Figure 5C), the mouse gut microbiota was dominated by *Muribaculaceae*, *Lachnospiraceae_NK4A136_group*, *Lachnospiraceae_unclassified*, *Prevotellaceae_Ga6A1_group*, *Desulfovibrio* and others. Among the top 20 genera, those closely associated with dietary fibre degradation and SCFA production were selected for between-group comparisons (Figure 5D–H). Although not all differences reached statistical significance, the MC group showed a significant increase in the relative abundance of *Candidatus_Saccharimonas* (*p* < 0.05) compared with the NC group, accompanied by decreases in *Prevotellaceae_Ga6A1_group*, *Ruminococcaceae* and *Odoribacter* (by 94.73%, 97.34% and 65.70%, respectively) and an abnormal increase in *Lactobacillus* (51.22%). Notably, PDMR intervention not only significantly reversed the aberrant increases in *Candidatus_Saccharimonas* and *Lactobacillus* (*p* < 0.05 in all intervention groups), but also further elevated the abundances of *Prevotellaceae_Ga6A1_group*, *Ruminococcaceae* and *Odoribacter*, restoring them to levels close to those of the NC group. These results indicate that PDMR can selectively modulate the abundance of specific SCFA-producing genera (such as *Lactobacillus* and *Ruminococcaceae*) and fibre-degradation-related genera (such as *Prevotellaceae_Ga6A1_group*), suggesting that these taxa may be key potential drivers mediating the regulatory effects of PDMR on lipid metabolism.

To identify the key gut microbiota with significant differences among groups, linear discriminant analysis effect size (LEfSe) was employed (LDA > 4, *p* < 0.05). First, compared with the NC group, the MC group was significantly enriched in *Candidatus_Saccharimonas* and *Clostridia_UCG-014* (Figure 5I), indicating a characteristic dysbiosis profile under obese conditions. Second, compared with the MC group, the HMR group was significantly enriched in *Alloprevotella*, *Ruminococcaceae*, *Prevotellaceae_Ga6A1_group*, *Oscillospiraceae_uncultured* and *Odoribacter* (Figure 5J), most of which are associated with SCFA production or fibre degradation. Notably, the aforementioned LEfSe results were fully consistent with the between-group variation trends of the five key genera described earlier, further confirming the selective regulatory effect of PDMR intervention on specific SCFA-producing functional microbial communities.

### 3.6. Effects of PDMR on the Metabolic Profile of Caecal Contents in Mice

To identify potential effector metabolites through which PDMR intervention ameliorates lipid metabolism at the metabolic level, untargeted metabolomics was performed on mouse caecal contents. Principal component analysis (PCA) score plots (Figure 6A) showed clear separation among the NC, MC and HMR groups (PC1 = 22.35%, PC2 = 16.85%), indicating that PDMR effectively improved the obesity-related metabolic profile. The partial least squares-discriminant analysis (PLS-DA) score plot (Figure 6B) further enhanced the between-group discrimination (Component 1 = 26.7%, Component 2 = 19.5%), and the corresponding permutation test confirmed the stability and good predictive ability of the PLS-DA model (Figure 6C).

To identify the specific characteristic metabolites regulated by PDMR intervention in lipid metabolism, differential metabolites were screened using the criteria of VIP > 1, *p* < 0.05 and fold change (FC) > 1.5 or < 0.667. As shown in the volcano plots (Figure 6D,E), 162 metabolites were significantly altered in the MC group compared with the NC group (99 up-regulated and 63 down-regulated). After PDMR intervention, 181 metabolites were significantly changed in the HMR group compared with the MC group (74 up-regulated and 107 down-regulated). Further focusing on the top 20 differential metabolites ranked by VIP score (Figure 6F,G), we found that the lipid-regulatory effects of PDMR were mainly reflected in the following key metabolite classes. Among these, bile acid metabolites were the most prominent category: secondary bile acids such as 3a,4b,12a-trihydroxy-5b-cholanoic acid, hyocholic acid, sodium deoxycholate and ursocholic acid were significantly elevated in the MC group (*p* < 0.05), whereas all showed significant downward trends in the HMR group after PDMR intervention (*p* < 0.05). In addition, vitamin D-related metabolites such as vitamin D3 sulfate and 26,26,26-trifluoro-25-hydroxy-27-norvitamin D3 were significantly decreased in the MC group (*p* < 0.05), and this phenomenon was significantly reversed in the HMR group (*p* < 0.05). Notably, inosine, a core intermediate of purine metabolism, was significantly enriched in the MC group (*p* < 0.05), suggesting that the high-fat diet may have induced an imbalance in energy metabolism. The plant-derived metabolite enterolactone was significantly enriched in the HMR group (*p* < 0.05), indicating that fermentation of PDMR by the gut microbiota may have produced bioactive polyphenolic metabolites.

KEGG enrichment analysis serves as an important bridge linking metabolite changes to biological functions, and its core value lies in mapping differential metabolites onto specific metabolic pathway networks, thereby revealing disease mechanisms and potential targets of interventions. Compared with the NC group, the high-fat diet significantly disrupted metabolic pathways including caffeine metabolism, purine metabolism, linoleic acid metabolism, lysine degradation and cysteine and methionine metabolism, which collectively contributed to high-fat-diet-induced obesity on a metabolic basis (Figure 6H). Compared with the MC group, PDMR intervention not only prominently regulated caffeine metabolism and linoleic acid metabolism, but also comprehensively restored purine metabolism and tryptophan metabolism, and additionally activated vitamin B6 metabolism (Figure 6I). Critically, linoleic acid metabolism and purine metabolism, two pathways closely associated with inflammatory regulation and energy homeostasis, were significantly enriched in both the MC and HMR groups. Taken together, the metabolomic results suggest that PDMR intervention may regulate gut microbiota-associated metabolites, particularly bile acids and purines, thereby influencing downstream inflammatory and energy metabolic pathways. This provides a plausible upstream–downstream causal link with the molecular observation that PDMR activates the hepatic AMPK pathway, as described earlier.

### 3.7. Correlation Analysis

To further explore the complex interactions amongst gut microbiota, differential metabolites and physiological indices, a correlation network was constructed based on Spearman’s rank correlation coefficients with false discovery rate (FDR) correction, using screening criteria of |r| > 0.6 and adjusted *p* < 0.05. As shown in Figure 7, *Oscillospiraceae_unclassified* was significantly negatively correlated with multiple bile acid metabolites, including hyocholic acid, sodium deoxycholate, ursocholic acid and 3-oxocholanic acid, as well as with the inflammatory receptor TLR4 (r < −0.8, *p* < 0.05); conversely, this bacterium was significantly positively correlated with the anti-inflammatory metabolite 24-hydroxyglabrolide (r = 0.83, *p* < 0.05). In contrast, *Candidatus_Saccharimonas* was significantly positively correlated with the aforementioned bile acid metabolites, LDL-C and the mRNA expression of the hepatic fatty acid synthase gene FAS (r > 0.7, *p* < 0.05). In addition, *Clostridia_UCG-014* was significantly positively correlated with TG (r = 0.82, *p* < 0.05) and significantly negatively correlated with UCP-1 (r = −0.78, *p* < 0.05). At the metabolite-phenotype level, 3a,4b,12a-trihydroxy-5b-cholanoic acid and ursocholic acid were both significantly positively correlated with LDL-C, TLR4 and IL-6 (r > 0.7, *p* < 0.05); hyocholic acid was significantly positively correlated with FAS (r = 0.73, *p* < 0.05) and significantly negatively correlated with the mRNA expression of the energy metabolism-related genes PGC-1α, PPARα and UCP-1 (r < −0.7, *p* < 0.05); and digitoxigenin was significantly positively correlated with TLR4 (r = 0.78, *p* < 0.05) and significantly negatively correlated with PGC-1α and UCP-1 (r < −0.7, *p* < 0.05). These results suggest that PDMR may ameliorate systemic inflammation and lipid metabolic disorders by remodelling the gut microbiota and reducing secondary bile acid-related pro-inflammatory or lipid-deposition metabolites.

## 4. Discussion

Obesity, one of the major threats to global public health, is characterised by a chronic imbalance between energy intake and expenditure, accompanied by dyslipidaemia, fat deposition and lipid metabolic disorders [26]. In recent years, meal replacement powders have attracted considerable attention as a convenient, quick and nutritionally valuable product for body weight management [28]. Our findings revealed that, in high-fat-diet-induced obese mice, PDMR intervention effectively attenuated body weight gain and reduced Lee’s index as well as the liver and spleen indices. Lee’s index is a classical indicator reflecting the degree of obesity and body fat accumulation, whilst the reductions in the liver and spleen indices suggest that PDMR may alleviate hepatic steatosis and inflammatory cell infiltration induced by a high-fat diet [34]. At the level of serum biochemical parameters, PDMR intervention significantly reduced serum TG, TC, LDL-C and FFA levels and increased HDL-C, indicating that PDMR can effectively reverse dyslipidaemia and metabolic disturbances in mice. Furthermore, the concomitant reductions in the pro-inflammatory cytokines IL-6, IL-1β and TLR4, along with the morphological recovery of adipocytes in epididymal adipose tissue, further indicate that PDMR not only alleviates systemic inflammatory responses but also ameliorates abnormal adipocyte hypertrophy. These phenotypic improvements indicate that PDMR, as a multi-component, high-dietary-fibre meal replacement powder, possesses a degree of lipid-lowering and lipid-regulating bioactivity. Unlike studies involving fibre supplements alone [37,38], this meal replacement powder intervention approach is therefore more practically relevant and better reflects real-world consumption patterns.

To elucidate the underlying molecular mechanisms by which PDMR ameliorates lipid metabolic disorders, the present study examined the expression of key proteins and genes in the AMPK pathway, a central energy sensor in the body. As a cellular energy sensor, AMPK is a critical regulator of cellular energy homeostasis; its phosphorylation and activation can simultaneously suppress lipid synthesis and promote lipid catabolism [39]. Our results demonstrated that PDMR treatment up-regulated the protein expression of p-AMPKα and p-ACC, whilst reducing the protein level of SREBP1c and the mRNA expression of FAS. The AMPK-ACC-SREBP1c axis represents a classical pathway for inhibiting de novo lipogenesis: AMPK phosphorylates ACC at Ser79, leading to its inactivation and a consequent reduction in the production of malonyl-CoA, a substrate for fatty acid synthesis, thereby relieving the inhibition of CPT-1-mediated fatty acid oxidation. Concomitantly, activated AMPK can inhibit the nuclear translocation of SREBP-1c, thereby suppressing the expression of key lipogenic enzymes, including FAS [40,41]. Therefore, modulation of this axis by PDMR directly inhibits excessive fatty acid synthesis in the liver, which is also a key factor contributing to the reductions in serum TG, TC and FFA levels.

In addition to suppressing lipogenesis, we observed that PDMR concurrently up-regulated the mRNA expression of PPARα and CPT-1, indicating enhanced fatty acid β-oxidation. CPT-1, as the rate-limiting enzyme for mitochondrial fatty acid uptake, is well established to be transcriptionally regulated by PPARα [42]. Moreover, the elevated mRNA expression of PGC-1α and UCP-1 indicated that PDMR promoted mitochondrial biogenesis and uncoupled thermogenesis. PGC-1α, a key activator of mitochondrial biogenesis, can synergistically enhance oxidative metabolism with PPARα, whilst UCP-1 uncouples mitochondrial oxidative phosphorylation from ATP synthesis through proton leakage, thereby dissipating energy as heat [43]. Notably, most existing studies have focused on the regulation of a single aspect of lipid metabolism [44,45]; by contrast, our study demonstrates that PDMR can bidirectionally regulate lipid metabolic disorders by, on the one hand, suppressing fatty acid synthesis through the AMPK-ACC-SREBP1c axis and, on the other hand, promoting fatty acid β-oxidation and thermogenesis through the AMPK-PPARα-CPT-1 and PGC-1α/UCP-1 axes, thereby exerting a synergistic regulatory effect on lipid metabolism in mice.

The gut microbiota serves as an important mediator between dietary components and host metabolism. Meal replacement powders, particularly those rich in dietary fibre, have been demonstrated to modulate the gut microbial ecology [27]. Our 16S rRNA sequencing results indicated that PDMR intervention improved the α-diversity and β-diversity of the gut microbiota in obese mice, with the HMR group exhibiting the highest number of unique OTUs, suggesting that PDMR can enhance microbial richness and reverse the high-fat-diet-induced disruption of gut microbiota structure. At the phylum level, PDMR reduced the F/B ratio. Substantial evidence has established that high-calorie diets cause an enrichment of *Firmicutes*, leading to an elevated F/B ratio, which in turn enhances the host’s capacity to extract energy from food and promotes fat accumulation [46]. The decrease in the F/B ratio therefore indicates that PDMR may reduce the efficiency of energy harvest by the gut microbiota, thereby lowering the risk of obesity. At the genus level, *Candidatus Saccharimonas* and *Clostridia_UCG-014* were significantly enriched in the MC group. Among these, *Candidatus Saccharimonas* has been shown in multiple studies to be positively associated with obesity, insulin resistance and systemic inflammation [47,48,49,50], suggesting that its enrichment in high-fat-diet-fed mice may contribute to the development of metabolic disorders. However, it should be noted that the role of *Clostridia_UCG-014* remains somewhat controversial: Zhou et al. [51] found that it was negatively correlated with LDL-C and TC, whereas other studies have reported that it can up-regulate inflammation-related indicators in animal experiments [52,53]. These inconsistent observations indicate that the functional impact of *Clostridia_UCG-014* may be context dependent, influenced by the broader microbial community structure and host metabolic status. Our correlation analysis showed that *Clostridia_UCG-014* was positively correlated with TG and negatively correlated with UCP-1, supporting its pathogenic association with high-fat-diet-induced obesity.

Furthermore, the HMR group was significantly enriched in genera associated with SCFA production and anti-inflammatory activity, such as *Alloprevotella*, *Ruminococcaceae*, *Prevotellaceae_Ga6A1_group* and *Odoribacter*. Among these, *Alloprevotella* is a Gram-negative obligate anaerobe capable of fermenting carbohydrates to produce SCFAs (acetate and butyrate) [54]. Multiple studies have demonstrated that its abundance is inversely associated with obesity, diabetes, cardiovascular diseases and metabolic syndrome [55,56]. *Ruminococcaceae* is a major butyrate-producing bacterial family; notably, there is heterogeneity at the species level, with *R. gnavus* often being associated with metabolic syndrome, type 2 diabetes mellitus and inflammation, whereas *R. bromii* ferments dietary fibre to generate SCFAs, thereby inhibiting lipogenesis and restoring insulin sensitivity [28,57]. *Prevotellaceae_Ga6A1_group* is not only an SCFA producer but was also found by Shen et al. [58] to be significantly enriched in a study of essential fatty acid regulation in type 1 diabetes, where its abundance was positively correlated with SOD activity. In addition, *Odoribacter* is also a common SCFA producer; moreover, Shi et al. [29] reported that increasing the abundance of *Odoribacter* could enhance resistance to intestinal pathogens, thereby repairing high-fat-diet-induced intestinal barrier damage. Collectively, these microbial changes suggest that PDMR, acting as a prebiotic preparation, may selectively enrich beneficial bacteria that produce SCFAs, exert anti-inflammatory effects and repair intestinal damage, whilst inhibiting harmful bacteria associated with obesity, inflammatory responses and metabolic disorders, thereby modulating the remodelling of the gut ecosystem and ameliorating lipid metabolic disorders.

Untargeted metabolomics results revealed how alterations in the gut ecosystem influence the host metabolic profile. In the present study, the regulation of differential secondary bile acid metabolites by PDMR—including 3a,4b,12a-trihydroxy-5b-cholanoic acid, hyocholic acid, sodium deoxycholate and ursocholic acid—is particularly noteworthy. Bile acids not only facilitate fat digestion and absorption but also act as potent signalling molecules that integrate metabolic, microbial and inflammatory pathways. Primary bile acids synthesised in the liver can be converted by the gut microbiota into secondary bile acids, which subsequently regulate host metabolism through activation of nuclear and membrane receptors, including the farnesoid X receptor (FXR) and the G protein-coupled receptor TGR5 [59]. However, high concentrations of secondary bile acids are cytotoxic, damaging the colonic epithelial barrier and inducing inflammatory bowel disease; they also inhibit beneficial carbohydrate-degrading bacteria (such as *Ruminococcus*, *Eubacterium* and *Bifidobacterium*) whilst enriching pro-inflammatory, protein-fermenting bacteria [60]. Furthermore, David et al. [61] found that a high-fat diet can perturb the gut ecosystem, leading to increased intestinal secondary bile acid concentrations and consequently promoting hepatocarcinogenesis. Our correlation analysis corroborates this, showing that 3a,4b,12a-trihydroxy-5b-cholanoic acid and ursocholic acid were both positively correlated with LDL-C, TLR4 and IL-6, whilst hyocholic acid was positively correlated with hepatic FAS mRNA expression and negatively correlated with hepatic PGC-1α, PPARα and UCP-1 mRNA expression. These correlations suggest that elevated secondary bile acids in obese mice may contribute to metabolic disorders by enhancing inflammatory signalling and inhibiting AMPK-driven fatty acid oxidation and thermogenesis, and that PDMR can effectively reverse this process.

KEGG enrichment analysis revealed that linoleic acid metabolism and purine metabolism were significantly enriched in both the MC and HMR groups, and both pathways are closely associated with inflammatory regulation and energy homeostasis, further underscoring the complexity of PDMR-mediated metabolic regulation. Linoleic acid and its metabolites have been implicated in systemic inflammatory responses and the development of atherosclerosis [62], whilst aberrant purine metabolism can lead to a hyperuricaemic state, which in turn induces systemic low-grade inflammation [63]. The observation that these pathways were enriched in both groups, yet with contrasting metabolic outcomes, suggests that PDMR may qualitatively shift the profile of differential metabolites within these pathways towards anti-inflammatory and energetically favourable directions, thereby ameliorating lipid metabolic disorders in the host.

To integrate these findings and construct a comprehensive regulatory mechanism, Spearman’s correlation analysis was conducted amongst gut microbiota, differential metabolites and host phenotypic indices. The correlation results strongly support the mediating role of the microbiota–metabolite axis in PDMR-mediated regulation of host lipid metabolism. Specifically, *Oscillospiraceae_unclassified* was significantly negatively correlated with multiple secondary bile acids and TLR4, whilst being positively correlated with 24-hydroxyglabrolide, suggesting that this bacterium may alleviate metabolic inflammation by reducing secondary bile acid production. In contrast, *Candidatus_Saccharimonas* was positively correlated with several secondary bile acid metabolites, LDL-C and FAS mRNA expression, indicating that this bacterium may induce lipid metabolic disorders by influencing bile acid metabolism and promoting fatty acid synthesis. Furthermore, digitoxigenin was negatively correlated with PGC-1α and UCP-1 mRNA expression, suggesting that this metabolite may suppress host energy expenditure. These correlation matrices reveal that PDMR may exert its anti-obesity effects through a potential gut microbiota- secondary bile acid–AMPK axis, improving systemic inflammatory responses and energy expenditure.

Based on these findings, the present study proposes a possible regulatory mechanism by which PDMR ameliorates lipid metabolic disorders: PDMR remodels the gut microbiota by enriching bacteria associated with SCFA-producing and anti-inflammatory activity (*Alloprevotella*, *Ruminococcaceae*, *Prevotellaceae_Ga6A1_group* and *Odoribacter*), whilst suppressing bacteria associated with pro-inflammatory and pro-adipogenic effects (*Candidatus_Saccharimonas* and *Clostridia_UCG-014*). The remodelled microbiota may further modulate host metabolic profile, reducing the excessive accumulation of secondary bile acids and thereby potentially activating the AMPK signalling pathway. This activation suppresses de novo lipogenesis through the ACC-SREBP1c-FAS axis, whilst promoting fatty acid β-oxidation and thermogenesis through the PPARα-CPT-1 and PGC-1α/UCP-1 axes, thereby synergistically ameliorating high-fat-diet-induced obesity and lipid metabolic disorders. This study not only holds considerable significance for the high-value utilisation of *Pyrus bretschneideri* Rehd. pomace, an agricultural by-product, but also provides a theoretical basis for its development as a functional food ingredient with anti-obesity properties.

This study provides insights into the lipid-lowering efficacy of PDMR and its potential mechanism of action; however, certain limitations remain. Firstly, although our correlation network revealed associations amongst gut microbiota, metabolites and host phenotypes, the causal relationships between specific bacterial strains and metabolic phenotypes still require verification through faecal microbiota transplantation or mono-colonisation experiments. Secondly, the bioactive components of PDMR are complex, comprising soluble fibre, insoluble fibre and bound polyphenols, and the specific active constituents and their individual effects need to be further isolated and identified. Third, food intake was not measured in this study, so the influence of differences in energy intake cannot be fully excluded. Future studies should employ isocaloric, macronutrient-matched and fibre-matched control diets or pair-feeding designs to clarify the relative contributions of pear pomace and other components of the formulation. Fourth, the sample size used for omics analyses in this study was relatively small; therefore, the microbiota and metabolomics results should be regarded as exploratory and hypothesis-generating in nature, and future validation in larger, adequately powered, independent cohorts, combined with functional experiments, is warranted. Finally, our study was conducted only in male mice; given the known sex differences in obesity pathophysiology and gut microbiota composition, future studies should also be performed in female mice.

## Figures and Tables

**Figure 1:**
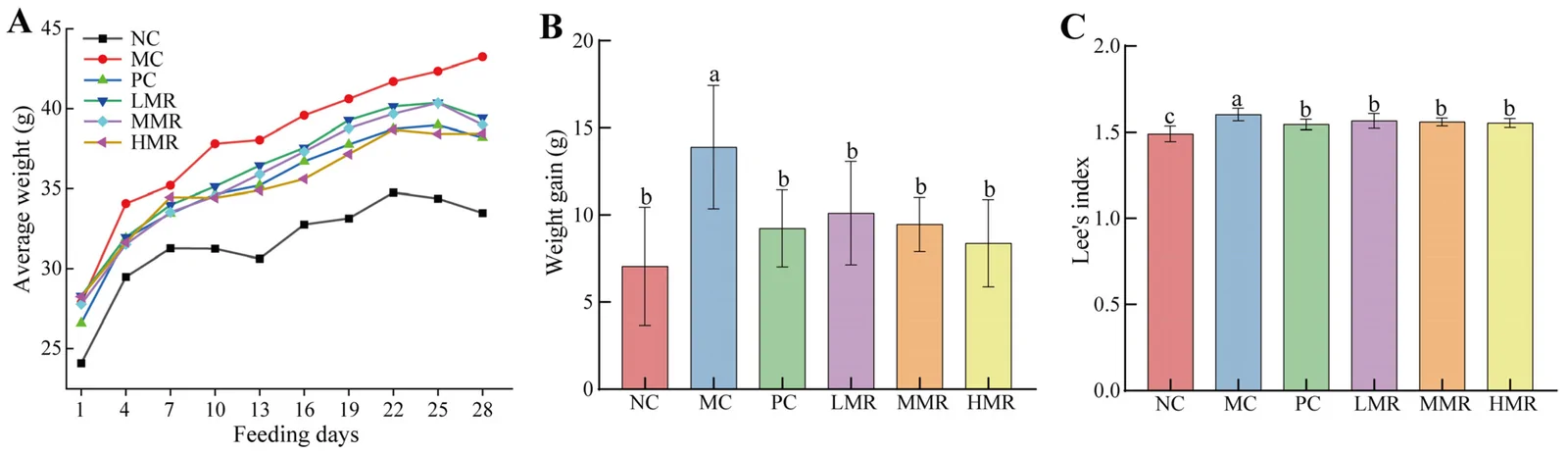
PDMR intervention ameliorates obesity in mice (*n* = 10). (**A**) Changes in body weight during the 28 d feeding period; (**B**) body weight gain; (**C**) Lee’s index. Different letters indicate significant differences (*p* < 0.05).

**Figure 2:**
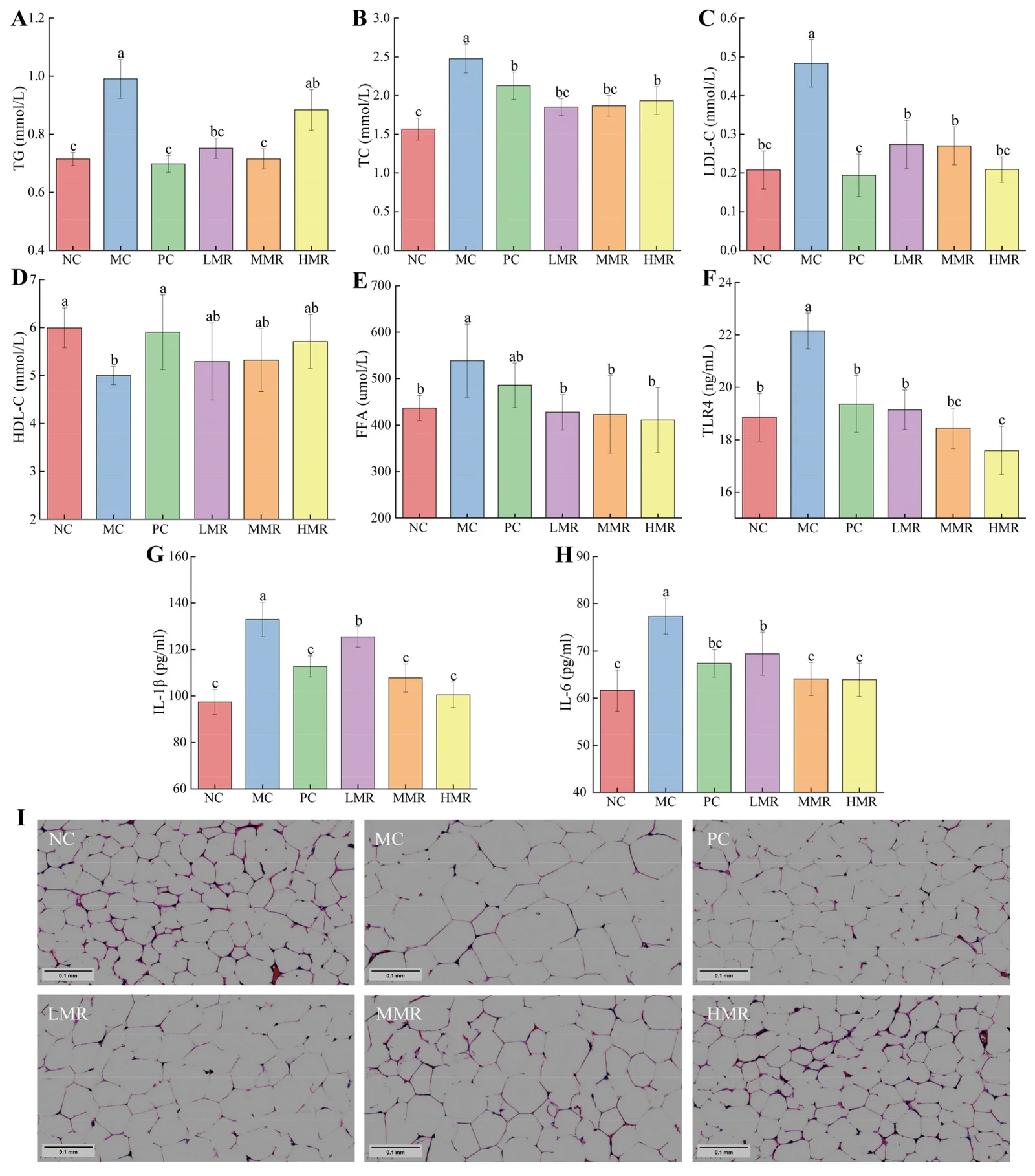
PDMR intervention restores physiological parameters in obese mice. Serum levels of TG (**A**), TC (**B**), LDL-C (**C**), HDL-C (**D**), FFA (**E**), TLR4 (**F**), IL-1β (**G**) and IL-6 (**H**) in mice (*n* = 10); H&E staining images of epididymal adipose tissue (20×, scale bar = 0.1 mm, *n* = 3) (**I**). Different letters indicate significant differences (*p* < 0.05).

**Figure 3:**
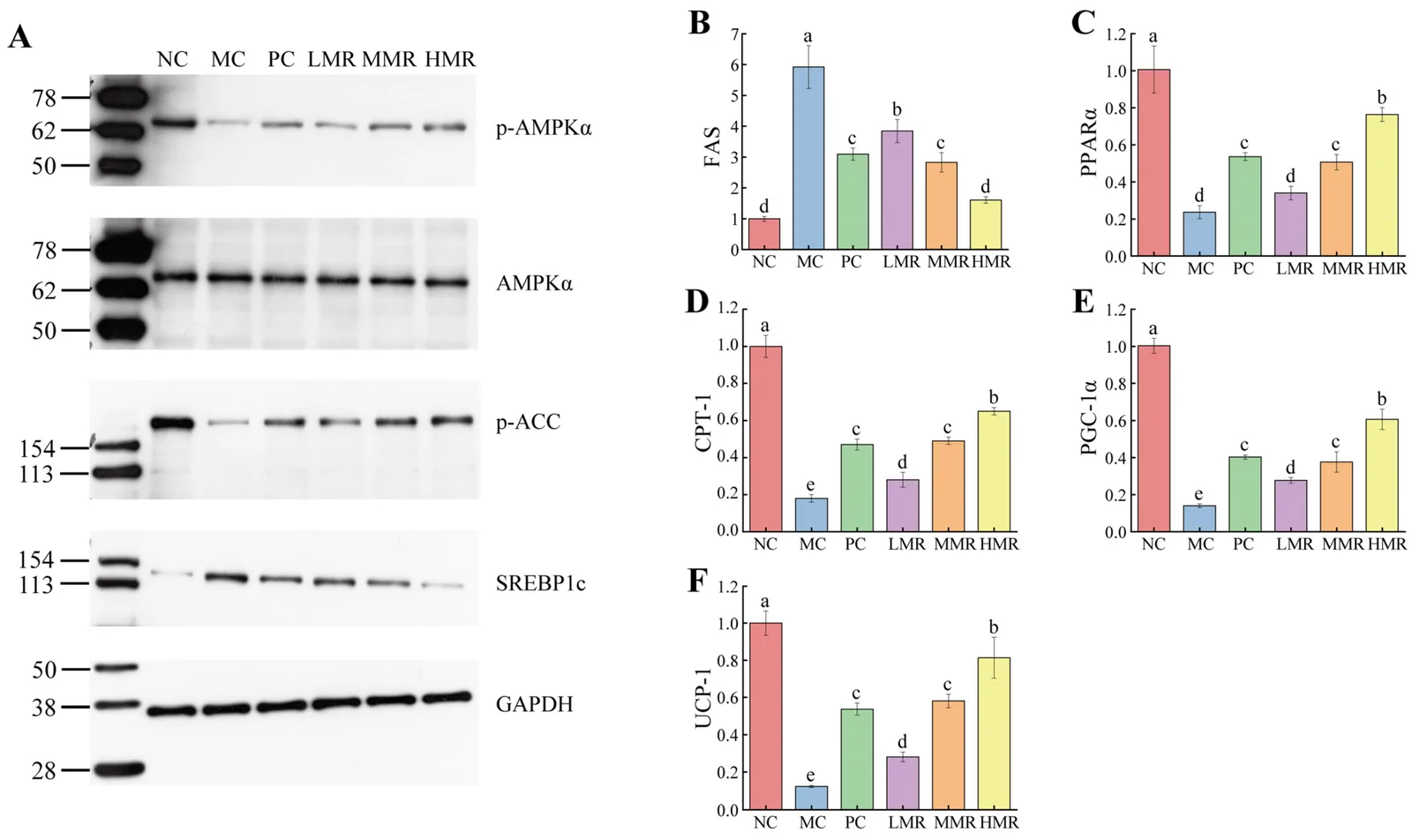
PDMR intervention regulates hepatic lipid metabolism in mice by activating the AMPK pathway (*n* = 3). Protein expression levels of p-AMPKα, AMPKα, p-ACC, SREBP1c and GAPDH in mouse liver (**A**); mRNA expression levels of FAS (**B**), PPARα (**C**), CPT-1 (**D**), PGC-1α (**E**) and UCP-1 (**F**). Different letters indicate significant differences (*p* < 0.05).

**Figure 4:**
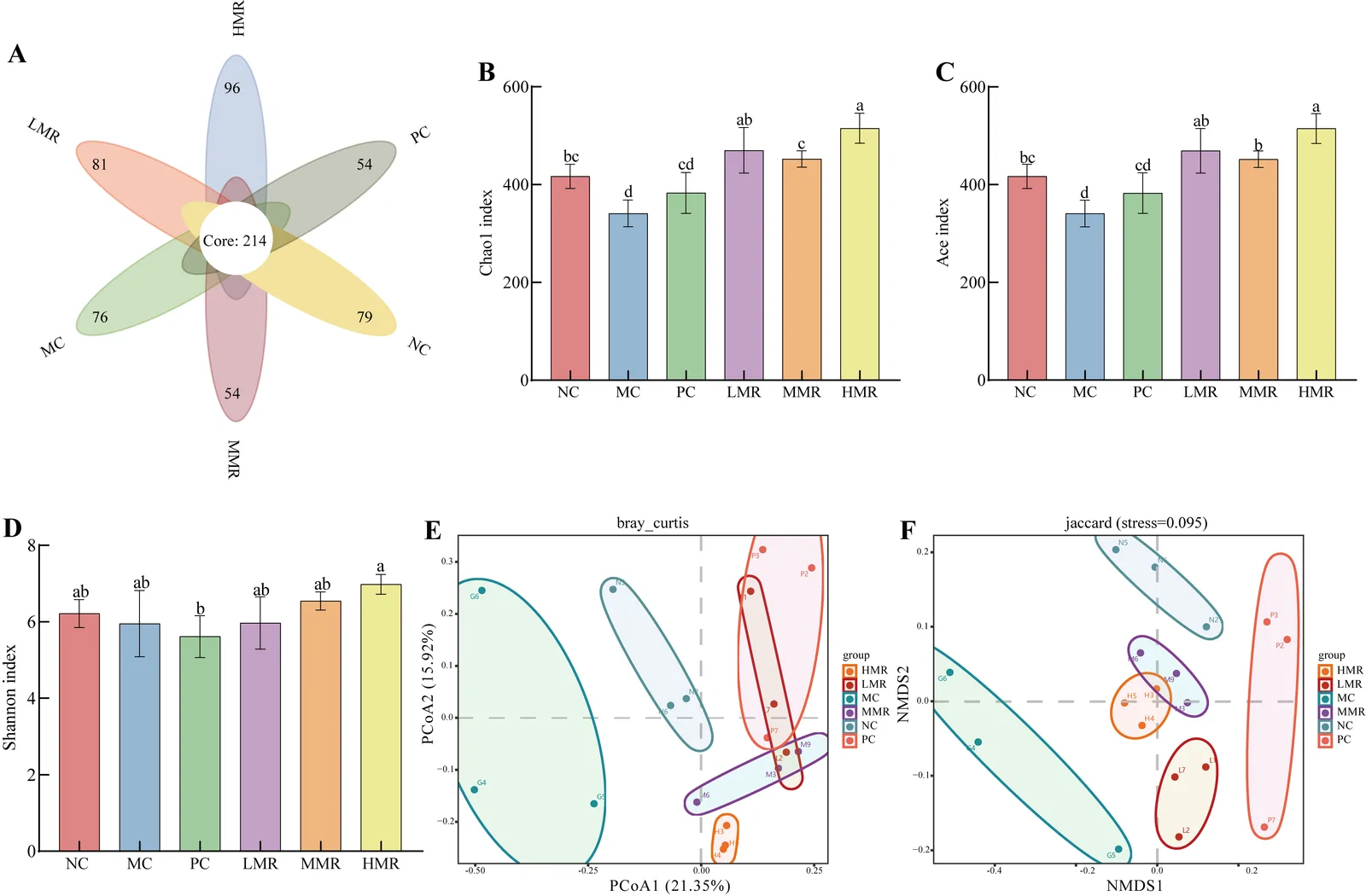
PDMR intervention improves gut microbiota diversity in obese mice (*n* = 3). Venn diagram of OTUs (**A**); Chao1 index (**B**); ACE index (**C**); Shannon index (**D**); PCoA analysis (**E**); NMDS analysis (**F**). Different letters indicate significant differences (*p* < 0.05).

**Figure 5:**
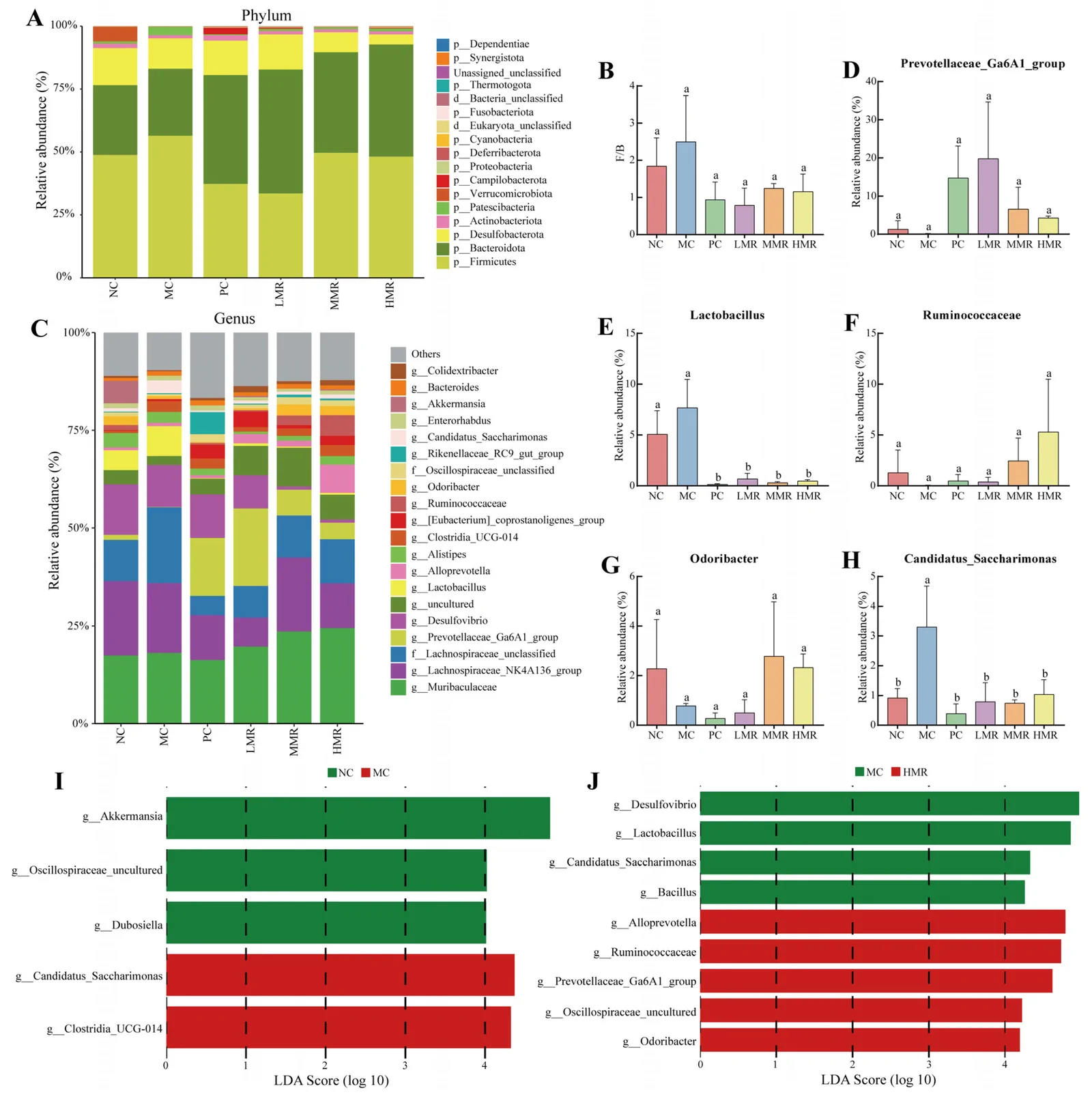
PDMR intervention reverses gut microbiota dysbiosis in mice (*n* = 3). Relative abundance of the gut microbiota at the phylum level (**A**); F/B ratio (**B**); relative abundance of the gut microbiota at the genus level (**C**); relative abundance of *Prevotellaceae_Ga6A1_group* (**D**), *Lactobacillus* (**E**), *Ruminococcaceae* (**F**), *Odoribacter* (**G**), *Candidatus_Saccharimonas* (**H**); LDA score plots for NC vs. MC (**I**) and MC vs. HMR (**J**). Different letters indicate significant differences (*p* < 0.05).

**Figure 6:**
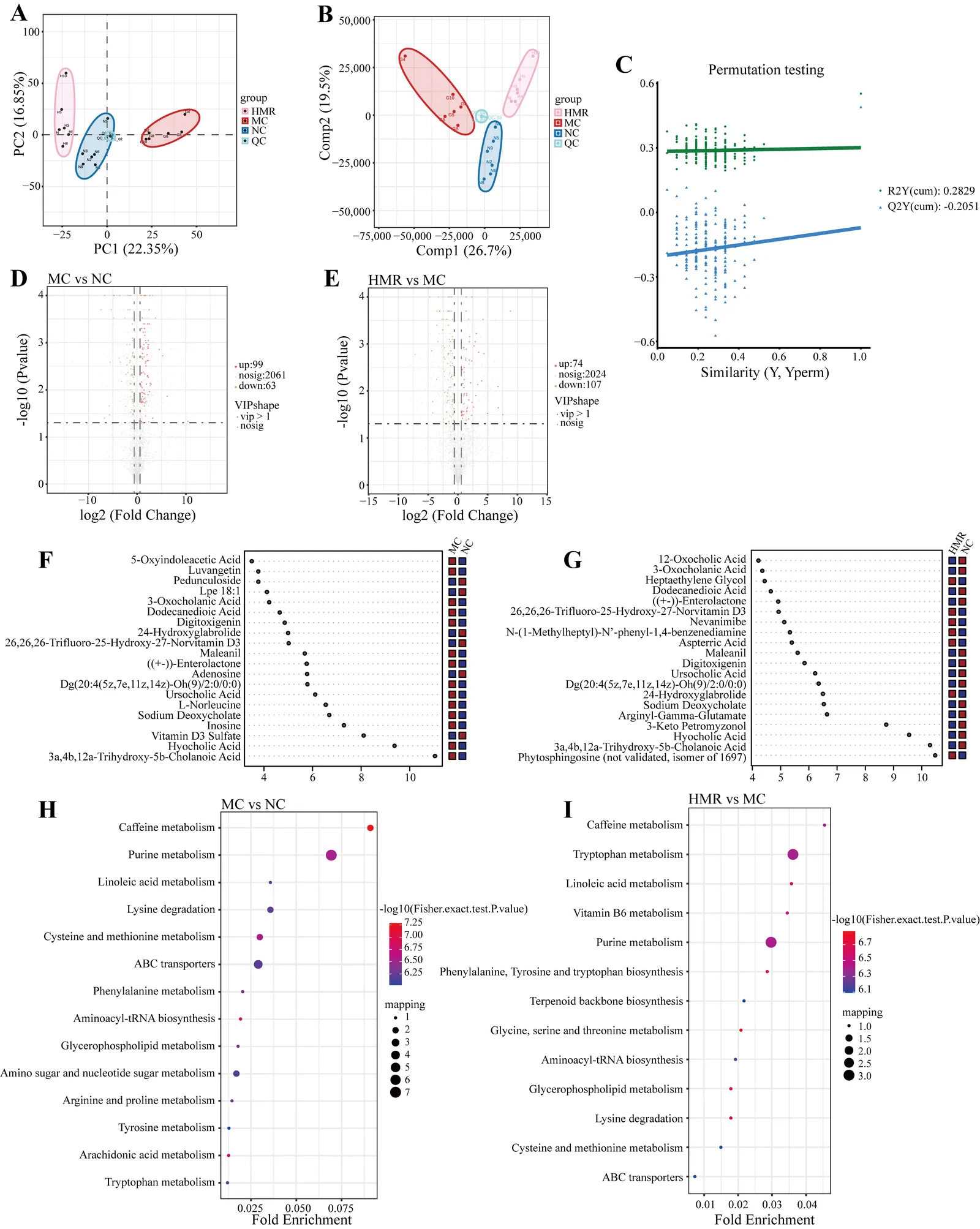
PDMR intervention ameliorates metabolic disorders in obese mice (*n* = 6). PCA score plot (**A**); PLS-DA score plot (**B**); PLS-DA permutation test (**C**); volcano plots of differential metabolites for NC vs. MC (**D**) and MC vs. HMR (**E**); VIP score plots of differential metabolites for NC vs. MC (**F**) and MC vs. HMR (**G**); bubble plots of KEGG pathway enrichment analysis of differential metabolites for NC vs. MC (**H**) and MC vs. HMR (**I**).

**Figure 7:**
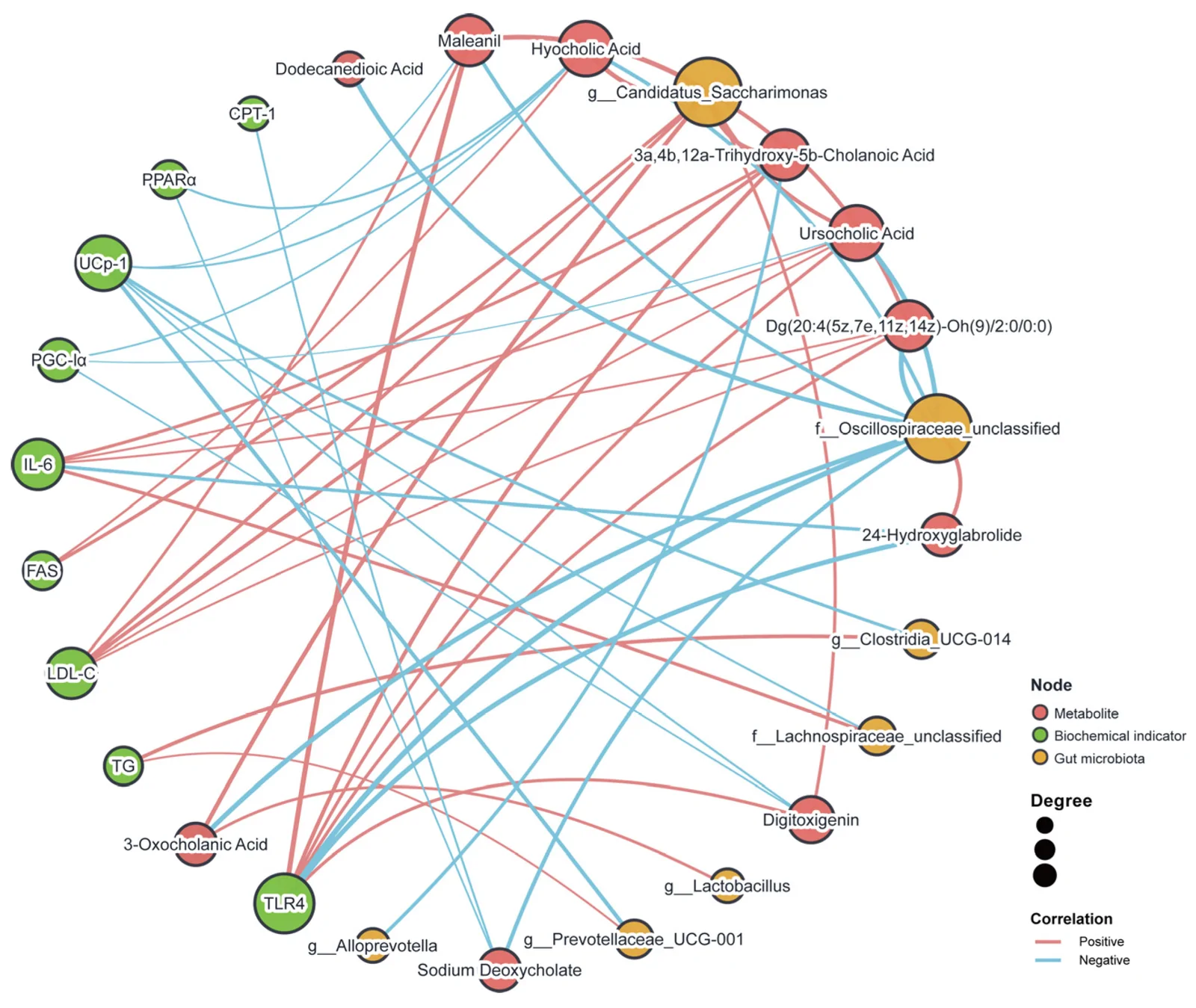
Correlation network amongst gut microbiota, differential metabolites and physiological indices under PDMR intervention. Thicker lines between nodes indicate stronger correlations.

**Table 1:** Primer names and sequences.

Primer Name	Sense/Antisense	Sequence (5′-3′)
M-GAPDH	Sense	TGAAGGGTGGAGCCAAAAG
	Antisense	AGTCTTCTGGGTGGCAGTGAT
M-CPT-1	Sense	CATGATTGCAAAGATCAATCGG
	Antisense	AGCACCTTCAGCGAGTAGCG
M-PPARα	Sense	CTCACAGAATTTGCCAAGGCT
	Antisense	CATAGCGAAGTCAAACTTGGGT
M-FAS	Sense	ATCTGGGCTGTCCTGCCTCT
	Antisense	TTATCAGTTTCACGAACCCGC
M-PGC-lα	Sense	GCAGGTCGAACGAAACTGACT
	Antisense	ACGCTGTCCCATGAGGTATTG
M-UCp-1	Sense	GCTTACAGAGTTATAGCCACCACAG
	Antisense	ACAGCTTGGTACGCTTGGGT

**Table 2:** Organ indices of mice in each group (*n* = 10).

Group	Heart Index/%	Liver Index/%	Lung Index/%	Kidney Index/%	Spleen Index/%
NC	0.507 ± 0.037 ^ab^	3.707 ± 0.241 ^bc^	0.618 ± 0.041 ^a^	1.089 ± 0.347 ^a^	0.251 ± 0.373 ^b^
MC	0.508 ± 0.050 ^ab^	4.759 ± 0.673 ^a^	0.622 ± 0.043 ^a^	1.159 ± 0.138 ^a^	0.297 ± 0.523 ^a^
PC	0.505 ± 0.053 ^ab^	3.401 ± 0.239 ^c^	0.616 ± 0.043 ^a^	1.088 ± 0.088 ^a^	0.265 ± 0.505 ^ab^
LMR	0.512 ± 0.037 ^a^	3.624 ± 0.243 ^c^	0.621 ± 0.046 ^a^	1.099 ± 0.065 ^a^	0.261 ± 0.439 ^ab^
MMR	0.515 ± 0.041 ^a^	3.612 ± 0.308 ^c^	0.627 ± 0.091 ^a^	1.102 ± 0.061 ^a^	0.264 ± 0.408 ^ab^
HMR	0.521 ± 0.026 ^a^	3.757 ± 0.186 ^b^	0.625 ± 0.022 ^a^	1.098 ± 0.106 ^a^	0.265 ± 0.268 ^ab^

Different letters indicate significant differences (*p* < 0.05).

## Data Availability

The original contributions presented in this study are included in the article. Further inquiries can be directed to the corresponding author.

## Abbreviations

The following abbreviations are used in this manuscript:

TG	Triglycerides
TC	Total cholesterol
LDL-C	Low-density lipoprotein cholesterol
FFA	Free fatty acid
HDL-C	High-density lipoprotein cholesterol
IL-6	Interleukin-6
IL-1β	Interleukin-1β
TLR4	Toll-like receptor 4
CPT-1	Carnitine palmitoyltransferase-1
PPARα	Peroxisome proliferator-activated receptor-α
FAS	Fatty acid synthase
PGC-1α	Peroxisome proliferator-activated receptor-γ coactivator-1α
UCP-1	Uncoupling protein-1
